# Antagonistic Interaction Between Microplastics and Herbivory on the Growth of Native and Invasive Plants

**DOI:** 10.3390/plants14172692

**Published:** 2025-08-28

**Authors:** Jeffrey Okundi, Ling Yuan, Guanlin Li, Daolin Du, Junmin Li

**Affiliations:** 1 School of Environment and Safety Engineering, Jiangsu University, Zhenjiang 212013, China; okundi2003@yahoo.com (J.O.); guanlinli1207@163.com (G.L.); 2 Zhejiang Key Laboratory for Restoration of Damaged Coastal Ecosystems, School of Life Sciences, Taizhou University, Taizhou 318000, China; lingyuan@tzc.edu.cn; 3 Zhejiang Provincial Key Laboratory of Plant Evolutionary Ecology and Conservation, School of Life Sciences, Taizhou University, Taizhou 318000, China

**Keywords:** microplastics, herbivory, biomass allocation, invasive plants, native plants, deleterious effects

## Abstract

Microplastic pollution and herbivory are increasingly recognized as significant stressors in terrestrial ecosystems, yet their interactive effects on native and invasive plants remain poorly understood. In this study, we investigated the individual and combined effects of polyethylene microplastics (PE-MPs) and herbivory by *Helicoverpa armigera* on the growth and functional traits of twelve plant species (six invasive and six native). Exposure to PE-MPs significantly reduced biomass accumulation, with larger reductions in shoot, root, and total biomass for native plants than for invasive ones. Herbivory also significantly reduced biomass accumulation. When combined, PE-MPs and herbivory produced antagonistic effects on shoot, root, and total biomass. No significant three-way interaction was found among PE-MPs, herbivory, and plant status. Both PE-MPs and herbivory significantly reduced the root mass fraction and root-to-shoot ratio (RSR) while increasing the shoot mass fraction, with the PE-MP-induced reduction in RSR being stronger in native plants. Our findings suggest that multiple anthropogenic stressors can act as ecological filters, reshaping plant competitive dynamics and accelerating community shifts toward stress-tolerant species.

## 1. Introduction

Microplastics, plastic particles smaller than 5 mm, accumulate in soils through agricultural practices, industrial waste, and urban runoff [1,2,3,4,5]. Growing evidence indicates that microplastic contamination is an emerging concern in terrestrial ecosystems, as it alters soil physical and chemical properties and affects biomass partitioning patterns [6,7]. As invasive species invade new areas, they often encounter microplastic-contaminated soils. Invasive plants increasingly encounter microplastic-contaminated soils in newly colonized habitats, yet it remains unclear whether their responses differ from those of native plants.

Microplastic contamination can differentially influence the growth performance of native and invasive plant species, thereby altering competitive dynamics and biomass allocation. Gentili et al. found that polyvinyl chloride (PVC) microplastics significantly reduced the biomass and photosynthetic efficiency of both *Senecio inaequidens* DC. and *Centaurea cyanus* L., indicating a general inhibitory effect on herbaceous species growth [8]. Similarly, Fu et al. observed that multiple microplastic types consistently suppressed aboveground biomass in both native and invasive plants, suggesting that greater microplastic diversity exacerbates physiological stress across taxa [9]. However, divergent responses emerge under varying environmental conditions. For example, Shi et al. found that native species exhibited stronger growth responses to microplastics under high-nutrient conditions, whereas invasive species maintained stable biomass through greater trait plasticity [10].

Such plasticity may allow invasive species to tolerate short-term stress, yet persistent soil contamination can still undermine their dominance, as shown in *Amaranthus palmeri* S. Watson communities [11]. Furthermore, Lozano and Rillig have reported that microplastics induced legacy effects on plant–soil interactions, disproportionately benefiting range-expanding (invasive) species by enhancing positive plant–soil feedbacks [12]. Collectively, these findings suggest that, although invasive species often possess a resilience advantage under microplastic stress, they may also suffer from disrupted competitive hierarchies and reduced long-term dominance. In contrast, native species may benefit from specific conditions (e.g., nutrient enrichment) but generally face greater physiological limitations and lower adaptive capacity. Thus, microplastic pollution constrains the growth of both groups and reshapes their ecological roles, potentially impeding native species recovery and destabilizing invasive plant communities.

Herbivory is a widespread biotic pressure that strongly influences plant fitness, structure, and competitive ability by causing biomass loss and altering allocation strategies [13]. It is another key stressor shaping plant performance and competition, often disadvantaging native species more severely than invasive ones. For instance, Geppert et al. found that native species in mountain ecosystems were more vulnerable to the combined effects of disturbance and herbivory, resulting in reduced biomass and competitive ability. In contrast, non-native plants better maintained their performance under these conditions [14]. This differential response suggests that herbivory can accelerate the decline of native species in disturbed habitats. Similarly, Sakata and Craig demonstrated that the presence of an exotic herbivore intensified competitive interactions, disproportionately favoring non-native plants by reducing the biomass of co-occurring native species [15]. Collectively, these studies indicate that invasive plants often show greater resilience or adaptive trait plasticity in response to herbivory, whereas native species frequently experience direct biomass losses and reduced competitive ability. Consequently, herbivory, particularly when coupled with other anthropogenic or ecological stressors, can enhance the competitive advantage of invasive plants while driving the decline of native plant communities.

Although the effects of microplastics and herbivory have been widely studied in isolation, their combined impact remains poorly understood. Evidence suggests that microplastics can alter the chemical composition of plants, including secondary metabolites and nutrient content. This may subsequently impact herbivore feeding preferences and damage severity [16,17]. Some studies have reported that microplastics can impair plant defense signaling pathways, such as those involving jasmonic acid, thereby reducing resistance to herbivores [18]. Conversely, microplastic-induced changes in leaf morphology, such as increased thickness or altered lignin content, have been shown to deter herbivore feeding or reduce larval development [19]. These outcomes appear to be species-specific, with invasive plants often exhibiting greater tolerance or compensatory growth, enabling them to better withstand dual stressors than native species. Exploring these combined effects could advance our knowledge of plant–herbivore interactions and inform strategies for invasive species management.

In this study, we conducted a controlled pot experiment to investigate the individual and combined effects of polyethylene microplastics (PE-MPs) and herbivory on biomass accumulation and allocation in native and invasive plant species. We aimed to determine (1) whether PE-MPs and herbivory act independently or interactively regarding plant growth and biomass allocation, (2) whether native and invasive species differ in their responses to these stressors, and (3) whether PE-MPs and herbivory alter biomass allocation patterns. Our findings offer insights into how multiple anthropogenic stressors may act as ecological filters, shaping plant competitive dynamics and potentially accelerating community shifts toward stress-tolerant species.

## 2. Results

### 2.1. Effect on the Biomass

**Figure 1:**
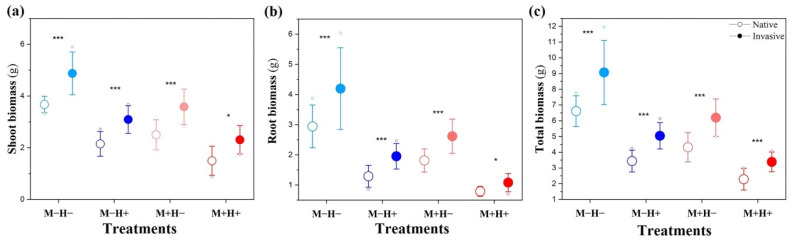
Effects of polyethylene microplastics (PE-MPs) and herbivory on shoot biomass (**a**), root biomass (**b**), and total biomass (**c**) of native and invasive plant species. The treatments include M−H− (no PE-MPs, no herbivory) (light blue color), M−H+ (herbivory only) (dark blue color), M+H− (PE-MPs only) (pink color), and M+H+ (combined PE-MPs and herbivory) (red color). Solid circles and hollow circles represent native and invasive species, respectively. Data are shown as mean ± standard error. Small dots represent outlier values. Asterisks indicate statistical significance (* *p* < 0.05; *** *p* < 0.001). Full statistical analyses of main and interaction effects are presented in Table 1.

Invasive plants had marginally significant higher shoot and total biomass than native plants (Figure 1a,c, Table 1). In addition, the effect of species nested within status was highly significant for shoot, root, and total biomass, indicating species-specific responses (Table 1).

Exposure to PE-MPs significantly reduced biomass accumulation, with shoot, root, and total biomass decreasing by 37.80%, 55.71%, and 45.72%, respectively (Figure 1a–c, Table 1). Significant interactions between plant status and PE-MPs were observed for root biomass and shoot biomass, but not for total biomass, indicating that PE-MP exposure led to a greater reduction in shoot and root biomass in native plants than in invasive ones (Figure 1a, Table 1).

Herbivory also significantly reduced biomass accumulation, with shoot, root, and total biomass decreasing by 27.88%, 39.42%, and 32.85%, respectively (Figure 1a–c, Table 1). No significant interaction was observed between herbivory and plant status for any biomass measure, indicating that both native and invasive species were similarly affected by herbivory (Figure 1a–c, Table 1).

The combined presence of PE-MPs and herbivory had an antagonistic effect on shoot, root, and total biomass, indicating that the combined stress was less severe than predicted by an additive model (Figure 1a–c, Table 1). For instance, shoot biomass decreased by 28.41% under herbivory alone (M−H− vs. M−H+), 38.30% under PE-MPs alone (M−H− vs. M+H−), and 54.97% under the combined treatment (M−H− vs. M+H+), which is less than the predicted additive reduction of 66.71%. No significant three-way interaction among PE-MPs, herbivory, and plant status was detected, suggesting similar effects on native and invasive species from these treatments.

### 2.2. Deleterious Effects (DEs) of PE-MPs and Herbivory

**Figure 2:**
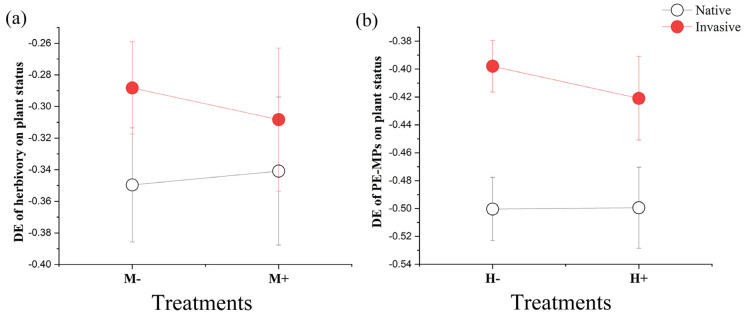
Deleterious effects (DEs) of herbivory (**a**) and polyethylene microplastics (PE-MPs) (**b**) on plant status in native (hollow circles) and invasive (solid circles) species. Data are shown as mean ± standard error. Full statistical results are provided in Table 2.

Neither PE-MPs nor plant status had a significant effect on the DE of herbivory based on total biomass (Figure 2a, Table 2), indicating that herbivory had a similar inhibitory effect on both native and invasive plants, and that the addition of PE-MPs did not modify this effect.

However, plant status significantly affected the DE of PE-MPs based on total biomass (Figure 2b, Table 2), with native plants experiencing greater damage than invasive ones. In addition, the effect of species nested within the status was highly significant, again indicating species-specific responses (Table 2). Moreover, there was no significant effect of herbivory on the DE of PE-MPs.

### 2.3. Effect on Biomass Allocation

**Figure 3:**
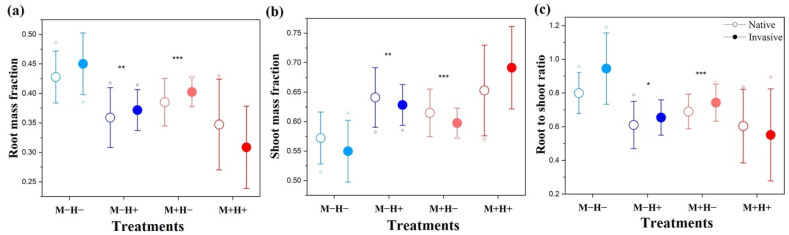
Line plots showing the main and interactive effects of polyethylene microplastics (PE-MPs) and herbivory on root mass fraction (**a**), shoot mass fraction (**b**), and root-to-shoot ratio (**c**) in native and invasive plant species. Treatments include M−H− (no PE-MPs, no herbivory) (light blue color), M−H+ (herbivory only) (dark blue color), M+H− (PE-MPs only) (pink color), and M+H+ (combined PE-MPs and herbivory) (red color). Circles and filled points represent native and invasive species, respectively. Data are shown as mean ± standard error. Small dots represent outlier values. Asterisks indicate statistical significance (* *p* < 0.05; ** *p* < 0.01; *** *p* < 0.001). Full statistical analyses of main and interaction effects are presented in Table 3.

Exposure to PE-MPs significantly reduced the root mass fraction (RMF) and root-to-shoot ratio (RSR) of plants, while increasing the shoot mass fraction (SMF) (Table 3, Figure 3). In addition, herbivory had the same effect (Table 3, Figure 3). No significant interaction between PE-MPs and herbivory was detected for RMF, SMF, and RSR (Figure 3, Table 3). However, a significant interaction between plant status and PE-MPs was found for RSR (Figure 3, Table 3). Specifically, PE-MPs significantly reduced the RSR of both invasive and native plants, with the reduction being stronger in native species.

## 3. Discussion

As an emerging pollutant, microplastics pose considerable risks to plant morphology, physiology, and growth [20]. Previous studies have shown that microplastic pollution can exert either negative or positive impacts on plant biomass, depending on plant species, and the shape and concentration of microplastics [12,21,22]. In this study, we found that exposure to PE-MPs significantly inhibited the biomass of both native and invasive plant species. Moreover, the reduction in shoot and root biomass induced by PE-MPs was greater in native plants than in invasive ones. Novel native generalist herbivores are common biotic pressures encountered by invasive plants in their invaded ranges [23]. Although invasive plants are more resistant to native generalist herbivores than exotic non-invasive congeners [24], native plants are generally more tolerant of such herbivores [25]. In our experiment, herbivory by the generalist herbivore *H. armigera* caused similar biomass reductions in native and invasive species, with no significant interaction with plant status. This similarity may result from the broad feeding behavior of the herbivore, which could overwhelm species-specific defenses, particularly in juvenile plants [14,26]. These results suggest that invasiveness may not necessarily confer resistance to biotic stress during the early stages of growth, when defenses are still developing [27,28].

There is a growing awareness that microplastic contamination of soils poses important threats to terrestrial ecosystems and can interact with other abiotic stressors, such as drought and UV-B radiation [21]. Contrary to our hypothesis, we found that the combined effects of PE-MPs and herbivory were antagonistic. Nguyen et al. have emphasized that the combination of abiotic and biotic stress did not always lead to additive effects, as unique molecular and biochemical responses can emerge under multifactorial stress conditions [29]. This aligns with the findings of Shan et al., who have reported that herbivory and elevated CO_2_ did not synergistically reduce biomass in either invasive or native species, suggesting that interactive stress effects were highly context-specific [30]. Taken together, these findings suggest that plants may engage in compensatory mechanisms, such as shifting resource allocation [31] or activating overlapping hormonal pathways [29], to buffer overall damage when PE-MPs and herbivory occur simultaneously. This antagonistic interaction suggests that the presence of one stressor may offset the impact of the other, potentially through compensatory physiological or resource allocation mechanisms. For example, herbivory may stimulate shoot regrowth to restore photosynthetic capacity, thereby offsetting the reduction in root biomass induced by PE-MPs [32,33]. Herbivory may specifically trigger systemic signaling that prioritizes carbon allocation to damaged aboveground tissue, thereby reducing the overall physiological burden imposed by microplastic-induced root stress [27,34]. Furthermore, PE-MPs can alter root traits and soil structure, influencing nutrient availability and hormonal signaling [19,35], which could modify plant defense responses. These soil-mediated changes could interfere with or suppress jasmonate-mediated defense pathways, which are commonly activated by herbivory, leading to reduced energetic trade-offs [13]. These findings underscore the complexity of plant responses in multi-stressor environments, highlighting the importance of considering non-additive interactions when assessing the ecological impact of combined environmental stressors.

Based on total biomass, we found that PE-MPs caused greater damage to native plants than to invasive ones. Similar patterns were reported by Li et al., who found that low-density polyethylene microplastic pollution induced greater biomass reduction in the native *Solidago* species than their invasive counterparts [22]. Invasive plants typically exhibited greater phenotypic plasticity in response to environmental changes than native plants did [36,37]. This would drive them to reallocate biomass to aboveground structures, enhancing their invasive abilities [37,38]. In our study, PE-MPs significantly reduced the RSR of both invasive and native plant species, with a greater decrease in the latter. This may be an adaptive strategy for invasive plants, allowing greater allocation to aboveground growth and enhancing fitness under microplastic stress [22]. Our results indicate that invasive species can buffer biomass allocation patterns, maintaining root–shoot functional stability despite environmental disruption. Invasive species, such as *Paspalum notatum* Flüggé, exemplify this by sustaining proportional investment in roots while maintaining shoot productivity, thereby securing both resource acquisition and competitive ability. Such allocation stability under stress aligns with evidence that adaptive biomass allocation contributes to invasion success, particularly in environments increasingly altered by anthropogenic pollutants [23,27,39].

Although some invasive species have shown resilience to microplastic pollution, our findings show that these responses are species-specific rather than determined by native or invasive status. For instance, the invasive species *P. notatum* exhibited the greatest tolerance of PE-MPs, while *Solidago canadensis* L. displayed greater sensitivity, highlighting variability even among invaders. Similarly, among native species, *Achyranthes bidentate* Blume was most sensitive, while *Lolium perenne* L. showed stronger tolerance. These patterns demonstrate that traits such as resource-use efficiency and allocation flexibility, rather than invasion status, could govern tolerance to microplastics. This implication is supported by the observations of Ali et al. [40]. Our findings support the idea that microplastics act as an abiotic ecological filter, favoring species with traits conferring resilience to altered soil conditions. Lozano & Rillig have consistently demonstrated that microplastic fibers can enhance the growth of invasive, range-expanding species such as *Calamagrostis epigejos* (L.) Roth, suggesting that certain invaders gain a direct growth advantage under microplastic stress [41]. Similarly, Li et al. have reported that invasive *S. canadensis* maintained biomass under low-density polyethylene (LDPE) microplastic exposure, while the native *S. decurrens* Lour. suffered significant reductions in both shoot and root biomass [22]. This aligns with our observation that species with high resource-use efficiency and allocation flexibility, traits often linked to invasiveness, performed better under PE-MP stress. Together, these findings reinforce the idea that microplastic contamination may drive shifts in plant community composition by preferentially favoring invasive, stress-tolerant species under global change scenarios.

## 4. Materials and Methods

### 4.1. Study Site and Plant Species Selection

This study was conducted in a controlled greenhouse at the Jiaojiang Campus of Taizhou University, Zhejiang, China. The region has a subtropical monsoon climate with an average annual temperature of 15–20 °C and annual precipitation of 1000–2000 mm (https://www.zjtz.gov.cn/col/col1229049312/index.html, accessed on 1 August 2024). The experiment aimed to evaluate the impact of microplastic pollution and herbivory on the partitioning of biomass in native and invasive plants.

Twelve plant species were selected for the study, including six invasive species and six native species (Table 4). Invasive species were chosen based on their documented ecological impacts and prevalence in local ecosystems, while native species were selected to match comparable growth forms and habitats for a balanced comparison. These species were classified as native or invasive based on The Checklist of the Alien Invasive Plants in China and validated using the Flora of China database. Seeds of *S. canadensis* (SC) were collected from natural populations near the Jiaojiang Campus of Taizhou University. Stem cuttings of *Wedelia trilobata* (Linn.) Hitchc. (WT) and *Wedelia chinensis* Merr. (WC) were provided by Jiangsu University in China and were subsequently propagated in a greenhouse at Taizhou University. Similarly, cuttings of *Alternanthera sessilis* (L.) R. Br. ex DC. (AS), *Alternanthera philoxeroides* (Mart.) Griseb. (AP) and *Hydrocotyl vulgaris* L. (HV) were obtained from a field near Taizhou University and transferred to a greenhouse for further growth. Seeds of *P. notatum* (PN), *Lolium perenne* L. (LP), *A. bidentata* (AB), *Solanum lyratum* Thunb. (SL), and *Crotalaria pallida* Aiton (CP) were obtained from the Thousand Green Seed Industry in China. Seeds of *Commelina communis* L. (CC) were obtained from the Dukou Renjia Seed Supermarket in China. The cutting stems and seeds were collected from the same population to avoid the potential of maternal effect. All of the plants were identified by Beifen Yang at Taizhou University.

### 4.2. Experimental Design

A factorial experimental design was employed to evaluate the impact of microplastic pollution and herbivory on plant biomass allocation. The study included three independent factors: microplastic treatment (soil containing 1% (*w*/*w*) PE-MPs versus control soil without PE-MPs); herbivory treatment (exposure to *Helicoverpa armigera* larvae versus control plants without herbivory); and plant status (invasive versus native). Each combination of these factors was applied to all twelve species, with five replicates per treatment group. This resulted in a total of 240 experimental pots (2 microplastic treatments × 2 herbivory treatments × 2 plant status categories × 6 species pairs × 5 replicates).

### 4.3. Seedlings Preparation

Seeds were planted on 5 August 2024, while stems from cuttings were transplanted on 26 August 2024. Peat moss (Pindstrup Plus, Ryomgaard, Denmark) was used as the growing substrate. The trays were arranged in the greenhouse under conditions of a day/night temperature of 25 °C/18 °C, 75% natural light intensity, and 60% humidity.

### 4.4. Microplastic Contamination Treatment

Polyethylene (PE), a common type of microplastic found in all environments and present in large quantities in sewage sludge used in agriculture [35], was selected for the study. The PE-MPs were obtained from Sinopec in China in powder form (100 mesh, excellent grade). Polyethylene pots (12 cm × 10.8 cm) filled with a 2:1 sand–soil mixture were used in this experiment. The soil was sourced from the mountain without microplastic contamination near Taizhou City. The soil had a pH of 4.53, 11.71 g kg^−1^ organic matter, 0.280 g kg^−1^ total nitrogen, and 1.206 g kg^−1^ total phosphorus. The PE-MPs were incorporated into the soil at a concentration of 1% (*w*/*w*), which corresponds to levels found in environmentally contaminated soils [38]. Each pot contained 800 g of soil, and the PE-MPs were thoroughly mixed into the substrate to ensure uniform distribution. Control pots contained identical substrate that was free from PE-MPs contamination. Trays were placed beneath all the pots to minimize water loss. The pots were arranged in the greenhouse.

### 4.5. Plant Transplanting

Seedlings were transplanted into pots on 17 September 2024 once they reached a uniform growth stage, determined by height, leaf number, and vigor. The initial plant height was measured and was taken as a covariate to eliminate the difference before stress treatments.

### 4.6. Herbivory Treatment

On 19 November 2024, i.e., five weeks after germination, herbivory stress was induced using third-instar *Helicoverpa armigera* larvae sourced from Jilin Haokang Biotechnology Co., Ltd. in Jilin, China. Two larvae were placed on each herbivory-treated plant’s young leaves using soft forceps. All herbivory-treated and control plants were enclosed in identical fine mesh cages (60 × 60 × 90 cm) to prevent larval escape and eliminate microclimatic variation. To maintain consistent feeding pressure, the second cohort of larvae was used again on 26 November 2024, and the third cohort on 3 December 2024. The final cohort of larvae was fed until 24 December 2024. Visual damage scoring was conducted after each replacement using a standardized scale (0~5, with 0 being the lowest and 5 being the highest) by the same observer to ensure the maintaining of consistent feeding pressure.

### 4.7. Harvest and Measurement

At the end of the twelve-week experiment, the plants were harvested and separated into shoot and root components. Both were then oven-dried at 70 °C for 72 h before being weighed. Total biomass was calculated as the sum of the dry weights of the roots and shoots.

To evaluate treatment-induced stress, we computed the deleterious effect (DE) for shoot biomass (DE_SB), root biomass (DE_RB), and total biomass (DE_TB) using the following formula: (Biomass treatment − Biomass control)/Biomass control. This formula quantifies the relative change in biomass due to the treatment compared with the control plants. A DE value greater than 0 indicates that the treatment facilitated plant growth, a value less than 0 indicates a reduction in growth, and a value of 0 reflects no effect. The more negative the DE value, the stronger the inhibitory effect of the treatment on plant biomass. This metric quantifies the proportional reduction in biomass caused by exposure to PE-MPs and/or herbivory, enabling standardized comparisons across treatments [44].

Additionally, the root-to-shoot ratio (RSR) was calculated by dividing root biomass by shoot biomass in order to assess biomass allocation patterns and to serve as an additional indicator of plant stress responses. Root mass fraction (RMF) and shoot mass fraction (SMF) were calculated as the proportion of total biomass allocated to roots and shoots, respectively. The formulas used are as follows: RMF = root biomass/total biomass, and SMF = shoot biomass/total biomass.

### 4.8. Statistical Analysis

All statistical analyses were performed using R version 4.2.1 [45]. Linear mixed-effects models were fitted using the lme function from the nlme package [46] to assess the effects of plant status (native vs. invasive), microplastic exposure (PE-MPs present vs. absent), and herbivory (present vs. absent), along with their two-way and three-way interactions, on total biomass, shoot biomass, root biomass, SMF, and RMF. Initial plant height was included as a covariate to account for differences in size prior to treatment.

To meet the assumptions of the model, the normality of the residuals was tested using the Shapiro–Wilk test. Where necessary, variables such as total biomass were square-root transformed. Plant species was included as a random effect to account for interspecific variation. Post hoc comparisons were conducted using the emmeans and pairs functions from the emmeans package to explore significant main effects and interactions. All tests were conducted with α = 0.05, and the results are reported as the mean ± standard error, unless otherwise stated.

Separate linear models were also used to evaluate the influence of plant status and stressor type (herbivory or microplastic exposure) on DE values. Post hoc comparisons were applied as needed to identify differential stressor impacts between plant groups.

## 5. Conclusions

Both PE-MPs and herbivory reduced plant biomass, with native species being more affected than invasive ones. Invasive species adjusted biomass allocation more flexibly and performed better under combined stress, suggesting an advantage in multi-stressor environments. The antagonistic interaction between PE-MPs and herbivory highlights the complexity of plant responses. These findings indicate that microplastic pollution may enhance the dominance of invasive species, posing risks to native plant communities, and have important implications for biodiversity conservation and ecosystem management.

## Figures and Tables

**Table 1:** Results of three-way ANOVA testing the main and interactive effects of polyethylene microplastics (PE-MPs), herbivory, and plant status on shoot, root, and total biomass of plants. Significant effects (*p* < 0.05) are indicated in bold.

Variable	Shoot Biomass		Root Biomass		Total Biomass	
	F	*p*	F	*p*	F	*p*
Initial height	25.313	**<0.001**	25.313	**<0.001**	40.450	**<0.001**
Plant status (S)	119.652	**<0.001**	119.652	**<0.001**	94.536	**<0.001**
Species (nested within status)	25.665	**<0.001**	25.665	**<0.001**	27.273	**<0.001**
PE-MPs (M)	177.389	**<0.001**	177.389	**<0.001**	285.106	**<0.001**
Herbivory (H)	85.679	**<0.001**	85.679	**<0.001**	125.359	**<0.001**
S × M	4.699	**0.031**	4.699	**0.031**	0.732	0.393
S × H	2.338	0.128	2.338	0.128	2.111	0.148
M × H	6.165	**0.014**	6.165	**0.014**	11.195	**0.001**
S × M × H	0.119	0.731	0.119	0.731	0.376	0.540

**Table 2:** Two-way ANOVA results testing the main and interactive effect of plant status and polyethylene microplastics (PE-MPs) on the DEs of herbivory, and those of plant status and herbivory (with vs. without) on the DEs of PE-MPs. Significant effects (*p* < 0.05) are indicated in bold.

Variable	DE of PE-MPs		De of Herbivory	
	F	*p*	F	*p*
Initial height	0.933	0.336	0.255	0.615
Plant status (S)	14.147	**<0.001**	1.756	0.188
Species (nested within status)	3.446	**0.001**	1.013	0.438
Herbivory (H)	0.223	0.638	/	/
PE-MPs (M)	/	**/**	0.013	0.910
S × H	0.274	0.602	/	/
S × M	/	/	0.152	0.698

**Table 3:** Results of three-way ANOVA testing the main and interactive effects of polyethylene microplastics (PE-MPs), herbivory, and plant status on biomass allocation traits, including root mass fraction, shoot mass fraction, and root-to-shoot ratio. The analysis evaluates both main effects and interactions. Significant effects (*p* < 0.05) are shown in bold.

Variable	Root Mass Fraction		Shoot Mass Fraction		Root-Shoot Ratio	
	F	*p*	F	*p*	F	*p*
Initial height	11.786	**0.001**	10.882	**0.001**	6.756	**0.010**
Plant status (S)	0.030	0.862	0.007	0.933	0.025	0.874
Species (nested within status)	7.201	**<0.001**	7.246	**<0.001**	6.290	**<0.001**
Microplastics (M)	27.274	**<0.001**	25.277	**<0.001**	16.003	**<0.001**
Herbivore (H)	9.894	**0.002**	8.888	**0.001**	5.232	**0.023**
S × M	3.104	0.080	3.360	0.068	4.104	**0.044**
S × H	0.066	0.798	0.503	0.852	0.023	0.880
M × H	0.455	0.503	0.167	0.683	1.107	0.294
S × M × H	1.061	0.304	1.055	0.306	0.810	0.369

**Table 4:** Information on the twelve herbaceous study species.

Species	Genus	Family	Number of Chinese Provinces	Status in China	Region of Origin	Characteristics	Mode of Reproduction
*Alternanthera sessilis*	Alternanthera	Amaranthaceae	14+	Native	Tropical Asia to America	Perennial, herb	Asexual
*Alternanthera philoxeroides*	Alternanthera		18	Alien-2	South America	Perennial, herb	Asexual
*Achyranthes* *bidentata*	Achyranthes		14	Native	Eastern and Tropical Asia	Perennial, herb	Asexual
*Solidago* *canadensis*	Solidago	Asteraceae	10+	Alien-3	North America	Perennial, herb	Sexual
*Wedelia* *chinensis*	Wedelia		5+	Native	Asia	Perennial, herb	Asexual
*Wedelia trilobata*	Wedelia		5+	Alien-2	Central America	Perennial, herb	Asexual
*Commelina* *communis*	Commelina	Commelinaceae	All except Qinghai/Xinjiang/Tibet	Native	Temperate Asia	Perennial, herb	Sexual
*Crotalaria pallida*	Crotalaria	Fabaceae	19	Alien-3	Tropical Africa and Tropical Asia	Annual, herb	Sexual
*Lolium perenne*	Lolium	Poaceae	North, South, Southeast China	Native	Europe	Perennial, grass	Sexual
*Paspalum* *notatum*	Paspalum		Widespread flowering in China	Alien	South America	Perennial, grass	Sexual
*Hydrocotyl* *vulgaris*	Hydrocotyle	Araceae	Yangtze River provinces (e.g., Zhejiang, Hubei)	Alien	Europe	Perennial, herb	Sexual
*Solanum* *lyratum*	Solanum	Solanaceae	Widespread in forests/disturbed sites	Native	East Asia	Perennial, herb	Sexual

Note: Overview of selected species and their biogeographic and ecological characteristics. Data on the distribution of each species across Chinese provinces were drawn from Yan et al. [42] for non-native species, and from Flora of China (http://www.efloras.org/flora_page.aspx?flora_id=2, accessed on 1 January 2024) for native taxa. The classification of alien species and their respective levels of invasiveness follows the framework proposed by Yan et al. [43], where Alien-2 indicates a high-impact invasive species, Alien-3 denotes localized invasions, and Alien-4 refers to widespread but low-impact aliens. Initial records of alien species in China were verified through the Chinese Virtual Herbarium (https://www.cvh.ac.cn/, accessed on 1 January 2024). Origin regions were determined using Plants of the World Online (POWO, 2019), and information regarding plant life cycles was obtained from Flora of China.

## Data Availability

The original contributions presented in this study are included in the article. Further inquiries can be directed to the corresponding author.

## Abbreviations

The following abbreviations are used in this manuscript:

PE-MPs	Polyethylene microplastics
DE	Deleterious effect
